# Ethik des langen Lebens

**DOI:** 10.1007/s00103-024-03866-w

**Published:** 2024-04-09

**Authors:** Hans-Jörg Ehni

**Affiliations:** Institut für Ethik und Geschichte der Medizin, Universität Tübingen, Gartenstr. 47, 72074 Tübingen, Deutschland

**Keywords:** Ageismus, Biogerontologie, Geroethik, Lebensverlängerung, Soziale Determinanten der Gesundheit, Ageism, Biogerontology, Geroethics, Life prolongation, Social determinants of health

## Abstract

Der Anstieg der durchschnittlichen Lebenserwartung, der sich seit 1850 bis heute weltweit vollzieht, kann als eine große zivilisatorische Errungenschaft gesehen werden. Viele sehen jedoch den demografischen Wandel und einen fortgesetzten Trend der steigenden Lebenserwartung über aktuelle Grenzen hinaus skeptisch. Die Gründe dafür liegen in kulturell tief verwurzelten Einstellungen zum Alter und zu älteren Menschen.

Dieser Beitrag stellt diesen Haltungen Grundsätze für ein langes Leben entgegen, die den Gewinn der ersten Revolution der Lebensverlängerung betonen. Forschung ist zu fördern, die diesen Gewinn sicherstellen kann und eine weitere Verlängerung der menschlichen Lebensspanne als Resultat einer zweiten Revolution in Aussicht stellt.

## Abstract (en)

The increase in average life expectancy that has taken place since 1850 and is continuing globally to the present day can be seen as a major achievement of civilization. However, many are skeptical about demographic change and the continuing trend of increasing life expectancy beyond current limits. The reasons for this lie in deeply rooted cultural attitudes towards old age and the elderly.

This article counters these attitudes with principles for a long life that emphasize the benefits of the first revolution of life extension. Research should be promoted that can ensure this gain and holds out the prospect of a further extension of the human lifespan as a result of a second revolution.

## Einleitung

Der Menschheit ist eine Revolution des langen Lebens gelungen. Seit 1850 ist die durchschnittliche Lebenserwartung, d. h. die statistisch zu erwartende Dauer eines Lebens ab der Geburt, in den in dieser Hinsicht am besten abschneidenden Staaten kontinuierlich jedes Jahr um etwa 3 Monate gewachsen. Die Gründe dafür liegen in verbesserten Lebensbedingungen, Hygiene, Ernährung und einer erfolgreichen Bekämpfung von Infektionskrankheiten. Dieser Anstieg der Lebenserwartung hat dazu geführt, dass global mittlerweile eine historisch noch nie erreichte Anzahl von Menschen erwarten kann, älter als 60 Jahre zu werden [1]. Die Prognosen darüber, wie sich diese Entwicklung fortsetzen wird, gehen auseinander. Manche Experten vermuten, dass in den Industriestaaten eine biologische Grenze erreicht sei [2]. Andere glauben dagegen, dass dieser demografische Trend fortgeführt wird. Die Hälfte der jetzt in Europa geborenen Menschen etwa könne erwarten, 100 Jahre alt zu werden [3]. Diese Entwicklung sehen manche mit Sorge oder Skepsis. Der vorliegende Beitrag untersucht die Gründe dafür und begegnet ihnen mit Grundsätzen für eine Ethik des langen Lebens.

## 75 Jahre sind genug!?

Verbreitet sind mittlerweile Einschätzungen, die den demografischen Wandel als Herausforderung sehen. Die Probleme wie wachsende Kosten im Gesundheitswesen oder Belastungen für die Rentenkassen scheinen zu überwiegen. Einen weiteren Anstieg der Lebenserwartung lehnen daher viele ab. Ein besonders interessantes Beispiel ist die Position des Medizinethikers und Onkologen Ezekiel Emanuel [4], der meint, dass 75 Jahre genug sind. Das soll nicht bedeuten, dass er mit 75 Suizid begehen will, sondern dass er jeder potenziell tödlich verlaufenden Erkrankung ihren Lauf lassen möchte, selbst wenn sie etwa durch Antibiotika einfach zu behandeln wäre. Seine Kritik richtet sich gegen einen Typus des Älterwerdens, den der „American Immortal“ verkörpern soll. Dieser „amerikanische Unsterbliche“ sei von Jugendlichkeit, Fitness und gesunder Ernährung besessen. Er könne die eigene Sterblichkeit nicht akzeptieren und wolle um jeden Preis länger leben.

Der Bedarf an unterschiedlichen Gütern, vor allem medizinischen, steigt mit dem Alter. Gleichzeitig nimmt die Produktivität ab. Man müsse sich also mit zunehmendem Alter fragen, ob der eigene Konsum noch durch die eigene Produktivität gerechtfertigt werde. Keinesfalls, so Emanuel, würden die durch die steigende Lebenserwartung gewonnenen Jahre auch in guter Gesundheit verbracht werden. Das Ziel einer „Morbiditätskompression“ sei ein Mythos. Dieser Begriff, den James Fries popularisiert hat [5], bezieht sich darauf, dass die Lebensspanne eingeteilt wird in eine gesunde Spanne („health span“) und eine Phase, in der chronische Erkrankungen auftreten. Letztere wird auch als „senescent span“ bezeichnet, wobei „Seneszenz“ die biologischen Alterungsprozesse meint, die zu altersassoziierten Erkrankungen führen oder zumindest für diese anfälliger machen sollen. Eine Morbiditätskompression liegt dann vor, wenn sich die seneszente Spanne zugunsten der Gesundheitsspanne verkürzt. Fries ging ursprünglich davon aus, dass sich lediglich die Anteile zwischen „health span“ und „senescent span“ verschieben, ohne dass die Lebensspanne insgesamt wächst. Tatsächlich ist jedoch die Lebenserwartung gestiegen und damit hat sich die gesamte Lebensspanne verlängert. Allerdings sei laut Emanuel dieser Anstieg nicht mit einer verbesserten Gesundheit verbunden. Eileen Crimmins Forschungsergebnissen für die USA zufolge sei mittlerweile das Gegenteil der Morbiditätskompression eingetreten, eine Morbiditätsexpansion. Allerdings ist laut Crimmins die Gesundheit im Alter deutlich differenzierter zu betrachten: Der Autorin zufolge ist eine Phase der Morbidität im hohen Alter kaum vermeidbar. Aber eine Morbiditätskompression sei durchaus bis 2001 bei Menschen mit höherem Einkommen zu beobachten [6]. Danach sank das Funktionsvermögen bei den unter 65-Jährigen, während Erkrankungen wie Diabetes mellitus und Adipositas in dieser Gruppe zunehmen – nicht bei Älteren – und ebenfalls in Abhängigkeit mit einem niedrigeren sozioökonomischen Status [7]. Es handelt sich also um Verluste von Funktionsvermögen, die durch Präventionsmaßnahmen vermeidbar sind.

Ähnlich einseitig sind Emanuels Behauptungen zu schöpferischen Leistungen im Alter, die er auf den Kreativitätsforscher Dean Keith Simonton stützt. Laut Emanuel sinkt die intellektuelle Produktivität nach einem Höhepunkt kontinuierlich ab, bis sie letztendlich ganz ausbleibt. Er bezieht sich dabei auf die Quantität von Beiträgen, die beispielsweise ein Wissenschaftler publiziert. Simonton hält jedoch fest, dass je nach Blickwinkel das Glas halbvoll oder halbleer sei [8]. Denn in vielen Bereichen bleibt die Anzahl der Publikationen bei der Hälfte dessen stehen, was sie am Höhepunkt der Leistungen war. Zusätzlich hängt aber dieser Höhepunkt nicht vom chronologischen Alter ab, sondern vom Zeitpunkt des Beginns und vom jeweiligen Bereich, in dem eine Leistung erbracht wird. So ist etwa die Philosophie für ein umfangreicheres Alterswerk ein günstigeres Betätigungsfeld als die Mathematik. Emanuels Behauptung, dass Altern ein Prozess sei, in dem geistige Produktivität kontinuierlich abnimmt, erweist sich als grobe bis irreführende Vereinfachung.

Emanuels Sicht auf das Alter als einen einseitigen körperlichen und geistigen Niedergang zieht schlüssig nach sich, dass die Älteren für die nachfolgenden Generationen mit zunehmendem Alter eine Last sind und ihnen Platz machen sollten. Dementsprechend sollte der Anstieg der durchschnittlichen Lebenserwartung in einer Gesellschaft ab einer bestimmten Altersgrenze nicht als Errungenschaft gesehen werden. Das Leben soll nicht um jeden Preis in jedem Zustand verlängert werden, so der Einwand. Das kommt ebenfalls im Motto „add life to years, not years to life“ (etwa „den Jahren Leben hinzufügen, nicht dem Leben Jahre“) zum Ausdruck, das sich die Gerontological Society of America bereits in den 1950er-Jahren gegeben hat. Laut Emanuel verkörpert der „American Immortal“ das Gegenteil dieses Mottos. Unter die Aktivitäten, die Emanuel angreift, fallen jedoch auch gesunde Ernährung und Sport, beides einem gesünderen Alter nach allgemeiner Ansicht zuträglich. Der Wunsch, das Leben um jeden Preis zu verlängern, ist nicht mit dem Wunsch gleichzusetzen, ein möglichst langes und gesundes Leben zu führen. Wer aber wie Emanuel die Lebensphase Alter als unvermeidlichen geistigen und körperlichen Niedergang sieht, wird beides gleichermaßen ablehnen. Hier zeigt sich deutlich, dass aus dieser Sicht der demografische Wandel ausschließlich ein Problem sei und ältere Generationen keinen positiven Beitrag zur Gesellschaft leisten. Emanuel wurde daher der Vorwurf der Altersfeindlichkeit und des Ageismus gemacht. Sein Standpunkt gehört zudem in die mit beidem verknüpfte Tradition des Apologismus.

## Apologismus

Apologismus bezeichnet nach dem amerikanischen Historiker Gerald Gruman die Verteidigung (Apologie) der menschlichen Existenz in ihrer jetzigen Form [9]. Er ist eine kulturelle und wissenschaftliche Position, die eine Verlängerung der menschlichen Lebensspanne weder für möglich noch für erstrebenswert hält. Den Gegensatz zum Apologismus nennt Gruman Prolongevitismus. Bereits in den 1960er-Jahren bezeichnete Gruman den Apologismus als mögliches Hindernis, Unterstützung für die durch den demografischen Wandel notwendige biomedizinische Forschung zu finden, welche ein gesünderes Alter ermöglichen könnte. Ein kultureller Apologismus rechtfertigt nach Gruman die Sinnhaftigkeit des „rauen Teils der Wirklichkeit“ („harsh realities“) der menschlichen Existenz wie Krankheit, Tod oder eben auch mancher negativen Erscheinungsformen des körperlichen Alterns. Damit ermöglicht er zwar, mit diesen Erfahrungen besser zurechtzukommen. Er verhindert jedoch gleichzeitig, dass sie verändert werden, sobald dies möglich sein könnte. Emanuels Text ist auch dafür ein gutes Beispiel. Emanuel lehnt zwar Forschungen zu einem gesünderen Alter nicht ab. Diesen schreibt er aber keine Priorität zu, da er argumentiert, dass die jetzige Lebensspanne genug sei, um das eigene Potenzial zu verwirklichen, und grundsätzliche psychische Grenzen dagegensprechen würden, dass dieses Potenzial sich auch bei körperlicher Gesundheit vergrößern würde. Der „American Immortal“ ist zudem eine Figur, die Grundmotive aus apologetischen Mythen vereinigt: Egoismus und Gier, fortschreitende Gebrechlichkeit und einen verhinderten Generationswechsel. Diese Motive lassen sich 3 sehr alten Erzählungen und Riten zuordnen: Egoismus und Gier dem Gilgamesch-Epos, die fortschreitende Gebrechlichkeit dem Tithonus-Mythos und der verhinderte Generationswechsel dem König des Waldes.

Gerald Gruman hält fest, dass apologetische Denkmuster in sehr alten historischen Mustern verwurzelt sind. Das Gilgamesch-Epos [10] erzählt, wie der Halbgott und König von Uruk Gilgamesch durch den Tod seines Freundes Enkidu von seiner eigenen Sterblichkeit beunruhigt wird. Gilgamesch macht sich auf die Suche nach dem einzigen Menschen, der durch die Götter Unsterblichkeit erlangt hat, Uta-Napischti, der babylonische Noah. Gilgamesch gelingt es zwar, diesen zu finden, aber sein Anliegen, unsterblich zu werden, wird zurückgewiesen. Er erhält jedoch den Hinweis auf eine verjüngende Pflanze. Nachdem er diese gefunden hat, schläft er erschöpft ein und unbewacht frisst eine Schlange das verjüngende Kraut. Gilgamesch kehrt nach Uruk zurück, erkennt die Vergeblichkeit seiner Bemühungen und sieht ein, dass seine Suche nach Unsterblichkeit ihn davon abgehalten hat, seinen Pflichten nachzukommen. Er nimmt von nun an seinen Platz ein und erfüllt seine Rolle. Das Streben nach Unsterblichkeit scheint im Gilgamesch-Epos vergeblich und auf Kosten anderer zu gehen. Die menschliche Gemeinschaft wird verlassen. Selbst ein Halbgott und herausragender Mensch wie Gilgamesch ist für die Unsterblichkeit nicht geeignet.

Auch der Tithonus-Mythos [11] erzählt von einer Beziehung zwischen Mensch und Gottheit, in der der Wunsch nach Unsterblichkeit scheitert. Tithonus ist der menschliche Geliebte der Göttin der Morgenröte, Eos. Er darf mit ihrer Fürsprache einen Wunsch aussprechen. Als er sich Unsterblichkeit wünscht, denkt er nicht daran, sich gleichzeitig ewige Jugend zu erbitten. Seine Unsterblichkeit ist daher mit einem immer weiter fortschreitenden körperlichen Alterungsprozess verbunden. Je nach Version des Mythos schließt ihn Eos am Ende schlicht weg oder verwandelt ihn in eine Grille.

Die dritte Figur, die bei Gruman fehlt, ist der König des Waldes. Sie entstammt einem römischen Ritus, der vom Anthropologen James Frazer in seinem Klassiker *The golden bough* (dt. *Der goldene Zweig*; [12]) analysiert wird. Der König des Waldes ist der Priesterkönig eines Dianatempels in der Nähe Roms und lebt in einem Hain. Wer einen goldenen Zweig von einem Baum dieses Hains bricht, kann den König des Waldes zu einem tödlichen Zweikampf herausfordern und, sofern er ihn besiegt, an seine Stelle treten. Frazer findet die einzelnen Elemente dieses Ritus in zahlreichen kulturübergreifenden Mythen wieder. Sehr knapp dargestellt lautet seine Interpretation der einschlägigen kulturellen Muster, dass bestimmten Personen magische Kräfte zugeschrieben werden, mit denen sie den Lauf der Natur zugunsten der jeweiligen menschlichen Gemeinschaft beeinflussen können. Sie sorgen für die Fruchtbarkeit und Erneuerung von Mensch und Natur, etwa indem sie gewährleisten, dass nach dem Winter der Frühling wiederkehrt. Verlieren diese Magier, Priester oder Könige ihre Kräfte durch Krankheit oder Alter, gefährdet dies die natürlichen Vorgänge, die sie beeinflussen können. Daher müssen sie regelmäßig ersetzt werden.

Der Apologismus warnt mit dem Scheitern von Gilgamesch und Tithonus vor dem Wunsch nach Unsterblichkeit, die für den Menschen nicht möglich ist und einen Ausdruck von Egoismus darstellt. Der Versuch, diesen Wunsch zu realisieren, wird lediglich mit einem fortschreitenden Verfall und dem Verlust der eigenen Menschlichkeit erkauft sowie auf der Ebene der Gemeinschaft mit der Stagnation, die der aufgehaltene Generationswechsel mit sich bringt. Gerade in diesem Wechsel der Generationen, der die Vitalität und Produktivität einer Gemeinschaft erhalten soll, liegt aus apologetischer Sicht einer der Sinngehalte des Alterns. Die zahlreichen negativen Altersstereotype, die damit verknüpft sind, werden sehr deutlich durch eine modernere Version der Figur des Tithonus veranschaulicht: Die Struldbrugs aus Jonathan Swifts *Gullivers Reisen* [13].

## Ageismus

Im dritten Teil von *Gullivers Reisen* erzählt Gulliver, wie er in Luggnagg erfährt, dass dort gelegentlich Unsterbliche geboren werden, erkennbar an einem Mal auf der Stirn. Gulliver gerät darüber zunächst ins Schwärmen, was er selbst als Unsterblicher an Weisheit und Reichtum erreichen und als kluger Ratgeber in der Gesellschaft bewirken könnte. Seine Gastgeber belehren ihn eines Besseren. Die Struldbrugs seien bösartige, geizige und gesellschaftlich isolierte, verachtete Geschöpfe, die wie Tithonus einem fortlaufenden physischen und psychischen Verfallsprozess ausgesetzt wären. Gulliver selbst beschreibt sie nach einer Begegnung als den hässlichsten Anblick, der ihm jemals geboten wurde. Er würde gerne einige Exemplare zurück nach England nehmen, wo sie als Mittel gegen die Todesfurcht gezeigt werden könnten. Interessant ist, wie Swift hier den Wunsch nach Unsterblichkeit mit der Negativität des Alters konfrontiert. Ein längeres Leben ist aufgrund der Eigenschaften, die ein höheres Alter notwendig mit sich bringt, nicht erstrebenswert. Der Tod ist gegenüber dem Alter das kleinere Übel und zu begrüßen. Hier wird deutlich, wie eine apologetische Argumentation mit negativen Altersstereotypen und Altersdiskriminierung verknüpft wird. Die Struldbrugs sind ein Kompendium von negativen Eigenschaften in Bezug auf Körper, Geist und Charakter, die dem Alter zugeschrieben werden. Diese Eigenschaften rechtfertigen eine gesellschaftliche Geringschätzung und Isolation. Es handelt sich um ein literarisches Paradebeispiel für Ageismus.

Der US-amerikanische Arzt und Altersforscher Robert Butler prägte im Jahr 1969 den Begriff „Ageismus“ [14]. Er stellte fest, dass die Ablehnung eines Wohnprojekts durch die Nachbarn vor allem damit begründet wurde, dass sie keine älteren Menschen in der Nähe wollten. Butler stellte fest, dass es sich um eine ähnliche Haltung handelte wie Rassismus oder Sexismus. Seitdem hat sich die gerontologische Forschung zunehmend mit diesem Phänomen beschäftigt und entsprechende Skalen und Messinstrumente entwickelt [15]. Internationale Organisationen (Weltgesundheitsorganisation (WHO), Vereinte Nationen), die ebenfalls die Bedeutung des globalen demografischen Wandels betonen, setzen sich mittlerweile ebenfalls vermehrt mit Ageismus auseinander. Einen Meilenstein markiert der umfangreiche Global Report on Ageism der WHO aus dem Jahr 2021 [16]. Ein gravierender Befund des Reports lautet, dass weltweit die Hälfte aller befragten Personen gegenüber älteren Menschen „ageist“ seien. Demnach seien Ältere (zu denen der WHO-Bericht Über-60-Jährige zählt) durchweg krank oder gebrechlich. Sie seien nicht offen für neue Erfahrungen bzw. nicht in der Lage, neue Kenntnisse und Fertigkeiten zu erwerben. Generell würden sie keinen Beitrag zur Gesellschaft leisten und seien vor allem eine Belastung.

Altersdiskriminierung und Ageismus sind nicht nur die Gründe von Benachteiligung und abwertender Beurteilung von älteren Menschen. Sie sind mit einer schlechteren körperlichen und psychischen Gesundheit verknüpft sowie mit einem niedrigeren allgemeinen Wohlbefinden. Eine kürzlich veröffentlichte Studie mit ca. 2000 Probanden zeigt eine Korrelation der Erfahrung von Ageismus im Alltag und zahlreichen Erkrankungen, wie etwa Herz-Kreislauf-Erkrankungen oder Diabetes mellitus [17]. Diese Faktoren wirken sich zudem stärker auf Personen mit niedrigerem sozioökonomischen Status (definiert durch Bildung, Einkommen, Arbeitsverhältnisse) aus – auf Gruppen also, die ohnehin gesundheitliche Unterschiede aufgrund von sozialen Determinanten der Gesundheit aufweisen. Ein gesünderes Alter erfordert daher eine ganze Bandbreite von Maßnahmen.

### Investitionen in die Lebensphase Alter

Wenn es um eine weitere Verlängerung der menschlichen Lebensspanne geht, rückt häufig die biologische Alternswissenschaft oder Biogerontologie (auf Englisch auch „Geroscience“ [18]) in den Mittelpunkt des Interesses. Die Schlagzeilen lauten dann: „Wann sind wir unsterblich?“ oder „Für immer jung“ [19]. Entsprechend große Aufmerksamkeit erhalten Forscher wie Aubrey de Grey, die propagieren, dass diejenigen schon geboren sind, die eine Lebensspanne von 1000 Jahren erreichen werden [20]. Diese Diskussionen über Altersutopien werfen interessante spekulative Fragen auf, lenken jedoch von konkreten Problemen und Lösungsvorschlägen für die nähere Zukunft ab. Im schlechtesten Fall wecken sie Bedenken durch hypothetische, radikale Umbrüche, die mindestens kurz- und mittelfristig nicht realisierbar sind.

Die Biogerontologie kann wahlweise in die Nähe des Transhumanismus oder der existierenden Anti-Aging-Medizin gerückt werden. Von ihren Gegnern wird ein (medizinisches) Eingreifen in das körperliche Altern abgelehnt, weil es sich um einen zu akzeptierenden, natürlichen Prozess handelt oder sogar für den Einzelnen nützlich sei. Eine solche Kategorisierung verkennt jedoch die zentralen Anliegen der biologischen Alternswissenschaft und einige ihrer zentralen Erkenntnisse zu körperlichen Alterungsprozessen. Es geht weder um einen kosmetischen Erhalt von Jugendlichkeit noch um eine radikale Verlängerung der menschlichen Lebensspanne. Exemplarisch für die Ziele der Biogerontologie und ihre Rechtfertigung kann man eine Gruppe von Autorinnen und Autoren um den bereits erwähnten Arzt Robert Butler und den Biologen S. Jay Olshansky heranziehen, die vor einigen Jahren gesellschaftliche Investitionen in die biologische Alternsforschung unter dem Schlagwort einer Langlebigkeitsdividende („longevity dividend“) gefordert haben [21]. Olshansky argumentiert, dass nach dem Erfolg der ersten Langlebigkeitsrevolution seit 1850 mithilfe von verbesserter Ernährung, Hygiene und der Behandlung von Infektionskrankheiten der Anstieg der durchschnittlichen Lebenserwartung bei der Geburt eine natürliche, biologische Grenze erreicht habe [22]. Dabei gebe es zwischen biologischer Alterung und altersbedingten Erkrankungen wie kardiovaskulären Erkrankungen, zahlreichen Krebserkrankungen und neurodegenerativen Erkrankungen einen engen Zusammenhang. Dieser Zusammenhang würde dafürsprechen, dass eine weitere Verbesserung der Gesundheit im Alter und ein weiterer Anstieg der durchschnittlichen Lebenserwartung ohne Therapien, die auf biogerontologischer Grundlage beruhen, nicht möglich sei. Bei Investitionen in diese biogerontologische Strategie stünde der Gesellschaft eine Langlebigkeitsdividende in Aussicht. Dabei seien in den nächsten Jahrzehnten beispielsweise 7 Jahre gesünderer, längerer Lebenserwartung realistisch und denkbar, was den Trend der ersten Langlebigkeitsrevolution fortsetzen würde. Schließlich sind teilweise die sozioökonomisch bedingten Unterschiede in der gesunden Lebenserwartung in zahlreichen Ländern größer als die 7 Jahre Langlebigkeitsdividende, die von den genannten Vertretern der Biogerontologie in Aussicht gestellt werden [23]. Es gibt folglich in beiden Kontexten einen ethischen Imperativ, in entsprechende Forschung und daraus abgeleitete Maßnahmen zu investieren.

## Grundsätze einer Ethik des langen Lebens

Aus diesen Überlegungen ergeben sich einige Grundsätze für eine Ethik des langen Lebens, die wie folgt angeordnet und erläutert werden. Die allgemeine Basis sowohl für den Einzelnen als auch für die Gesellschaft muss die Wertschätzung des langen Lebens selbst darstellen. Damit individuell ein langes Leben ebenfalls ein gutes Leben ist, sollten einige Klugheitsregeln berücksichtigt werden. Diese stellen als solche keine moralischen Verpflichtungen, sondern Empfehlungen dar, sofern man sich die entsprechenden Ziele zu eigen macht. Auf gesellschaftlicher Ebene gibt es die moralische Verpflichtung, Rahmenbedingungen zu schaffen, die für unterschiedliche Lebensentwürfe eines langen Lebens notwendig sind. Um vor Diskriminierung bei moralischer Gleichheit zu schützen, ist auf gesellschaftlicher Ebene Apologismus kritisch zu hinterfragen und Ageismus zu bekämpfen. Schließlich ergeben sich aus der Forderung der Chancengleichheit für verschiedene Lebensentwürfe eines langen Lebens Ansprüche des Einzelnen darauf, dass gesellschaftlich die notwendigen Rahmenbedingungen zugänglich gemacht werden, die unabhängig vom sozioökonomischen Status ein langes Leben ermöglichen, wie beispielsweise fortgesetzte Bildung, soziale Teilhabe, Gesundheitsversorgung und Erwerbstätigkeit. Zu den Grundsätzen im Einzelnen:

### Die Wertschätzung für ein langes Leben fördern

Da ein langes Leben trotz Apologismus kulturübergreifend geschätzt wird, könnte man meinen, dass dieser erste Grundsatz überflüssig sei. Teils wird jedoch sogar wie im oben zitierten gerontologischen Leitsatz nahegelegt, die Länge des Lebens spiele keine Rolle oder es sei besser, wenn das Leben kurz, aber intensiv sei. Der demografische Wandel nährt vollends die Skepsis daran, ob ein langes Leben oder eine weitere Lebensverlängerung zivilisatorische Errungenschaften verkörpern. Auch hierfür ist Emanuels Aussage beispielhaft, man solle die Lebenserwartung ab einer gewissen Grenze nicht mehr als Kriterium für den Erfolg einer Gesellschaft verwenden. Gleichzeitig stellt der Wert des menschlichen Lebens einen Grundwert unserer Gesellschaft dar. Er gilt zwar nicht absolut, etwa gegenüber der Selbstbestimmung, jedoch kann man offensichtlich nicht behaupten, es gebe einen Konsens, dass das Leben ab einer gewissen Lebensspanne kein Gut mehr sein soll oder man tödlichen Erkrankungen dann regelmäßig ihren Lauf lassen sollte. Im Gegensatz dazu werden etwa sozial bedingte Unterschiede in der Lebenserwartung als ungerecht eingestuft ebenso globale Unterschiede zwischen Ländern mit hohen, mittleren oder niedrigen Einkommen. Diesen Bewertungen liegt zugrunde, dass Lebenszeit als gerechtigkeitsrelevant eingeschätzt wird.

### Alter als Teil des langen Lebens wertschätzen

Zur Wertschätzung des langen Lebens gehört auch die Wertschätzung der Lebensphase Alter, einschließlich des hohen Alters. In diesem Zusammenhang müssen die Kontinuität des Lebens, die Vieldeutigkeit des Alterns und die Heterogenität älterer Menschen hervorgehoben werden. Es gibt keine einfachen chronologischen Grenzen, durch die sich Lebensphasen abtrennen lassen. Zurückzuweisen sind auch die traditionellen Stufenmodelle, denen zufolge das Leben aus einem Aufstieg, Höhepunkt und Abstieg besteht. So steigt beispielsweise laut vielen Studien die subjektive Zufriedenheit mit dem Alter an, nachdem sie in der mittleren Lebensphase statistisch einen Tiefpunkt erreicht hat. Auch wenn die gerontologische Forschung das hohe Alter durch eine Zunahme von Verletzlichkeit charakterisiert sieht, nimmt man auch hier an, dass Wachstum möglich und eine reine Verlustsicht auf die späte Lebensphase nicht gerechtfertigt ist [24].

### Das lange Leben gestalten

Ein langes Leben und das Alter wertzuschätzen bedeutet auch, es zu gestalten. Wenn die Lebenserwartung weiter steigt und die längere Lebensspanne mit erfüllender Tätigkeit verbracht werden soll, steigt die Bedeutung eines Lebensstils, der sich der längeren Lebenszeit bewusst ist. Das gilt für einen Lebensstil, der die Gesundheit erhält, ebenso wie für ein Bewusstsein für Tätigkeiten und Fähigkeiten, die für ein längeres Leben nützlich sind, wie etwa Offenheit für Neues, Bildung und auch die Bereitschaft, zu wechseln [25]. Bereits 1991 hat der Soziologe Peter Laslett in seinem Buch *A fresh map of life* darauf hingewiesen, dass der Anstieg der Lebenswartung zu einer neuen Lebensphase geführt hat [26]. Man müsse sich von der schlichten Einteilung des Lebens in die „three boxes of life“, also Ausbildung, Berufstätigkeit und Pension, verabschieden und mehr Kontinuitäten und Entwicklung erlauben, wie etwa ein lebenslanges Lernen. Das Motto muss also lauten: „Add life to years AND years to life.“

### Kulturelle und gesellschaftliche Rahmenbedingungen für ein langes Lebens schaffen

Die individuelle Gestaltung des langen Lebens und des zukünftigen Alters erfordert eine notwendige Infrastruktur, wie Weiterbildungsmöglichkeiten, passende Gelegenheiten (etwa für soziales und ehrenamtliches Engagement) und die nötigen gesellschaftlichen Ressourcen. Hierzu gibt es zahlreiche Stellungnahmen und Berichte auf nationaler und internationaler Ebene. Ebenso grundlegend sind jedoch die kulturellen Ressourcen, die als Ausdruck gesellschaftlicher Wertschätzung des langen Lebens und des Alters ebenso wichtige, elementare Rahmenbedingungen darstellen. Diese Wertschätzung kann nur dann zum Ausdruck kommen, wenn die unterschiedlichen Formen des Apologismus hinterfragt werden und der damit verknüpfte Ageismus bekämpft wird. Insbesondere der negative Apologismus ist damit gemeint, der im Wunsch nach einem längeren Leben Gier sieht, Altern ausschließlich als Verlust und der zur gesellschaftlichen Erneuerung einen Generationswechsel als notwendig behauptet. Interessanterweise nennt die Antidiskriminierungsstelle des Bundes (ADS) in ihrem Bericht zu Ageismus unter den problematischen Ansichten zuerst: „Ältere Generationen sollen jüngeren Platz machen“, die von etwa 30 % der Befragten geteilt wird [27]. Die Alternative besteht in der Kooperation und im Austausch zwischen den Generationen [28]. In *The generation myth* beschreibt der Soziologie Bobby Duffy, wie dieser Austausch unter anderem durch unterschiedliche technisch-kommunikative Lebenswelten gelitten hat. Da wir aber alle nacheinander das lange Leben durchlaufen und sowohl die Probleme als auch die Chancen generationenübergreifend sind, gibt es keine Alternativen zur intergenerationellen Solidarität und zur Bekämpfung von Generationsmythen und Stereotypen über die unterschiedlichen Generationen hinweg. Diese ersten 4 Grundsätze auszuformulieren, gehört zu den Kernaufgaben einer Ethik des Alter(n)s oder Geroethik, die philosophisch ebenfalls weiterzuentwickeln ist [29].

### Forschung fördern, die dem langen Leben dient

Schließlich kann die größere gesellschaftliche Wertschätzung des langen Lebens und der Lebensphase Alter die Bereitschaft vergrößern, wissenschaftlichen und technischen Fortschritt zu unterstützen, der beidem zugutekommt. Biogerontologische Erkenntnisse und ihre Anwendung können für ein gesünderes Altern bedeutsam sein, wenn verlangsamtes Altern auch bedeutet, dass altersbedingte Erkrankungen verhindert werden und die entsprechende Morbidität tatsächlich komprimiert wird, wie es Tierversuche nahelegen. Weitere Chancen bietet die Digitalisierung, unter anderem auch als Unterstützung bei unvermeidbarer Vulnerabilität [30].

Die dargestellten Grundsätze greifen ineinander. Sie bejahen individuell und gesellschaftlich eine zweite Revolution der Langlebigkeit und den Gewinn, den eine weitere Verlängerung des menschlichen Lebens darstellen kann. Auf der Basis der Wertschätzung des langen Lebens sollen kulturelle Denkmuster und Vorurteile geändert werden. So kann die Bereitschaft gefördert werden, die nötigen Ressourcen für die neue Zukunft des Alters bereitzustellen. Der neue demografische Wandel, der daraus entstehen kann, wäre in der Tat eine weitere zivilisatorische Errungenschaft.
